# 666. Evaluation of Vancomycin Harms for Intra-abdominal Infections

**DOI:** 10.1093/ofid/ofad500.729

**Published:** 2023-11-27

**Authors:** Christopher McCoy, Xuping Yan, Ryan W Chapin, Kendall Donohoe

**Affiliations:** Beth Israel Deaconess Medical Center, Boston, Massachusetts; Mount Auburn Hospital, Boston, Massachusetts; Beth Israel Deaconess Medical Center, Boston, Massachusetts; Beth Israel Deaconess Medical Center, Boston, Massachusetts

## Abstract

**Background:**

The Infectious Diseases Society of America recommends broad spectrum Gram-negative and anaerobic coverage for complicated intra-abdominal infections (IAI). Gram-positive beta lactam resistant organisms such as methicillin-resistant *S. aureus* (MRSA) are not typically associated with pathogenicity. Vancomycin, an empiric Gram-positive agent, is associated with nephrotoxicity. This study evaluated incidence of harm in patients treated with antimicrobial regimens which included vancomycin vs. those which did not.

**Methods:**

This single center, retrospective cohort study included adult inpatients admitted between September 1, 2018 and September 30, 2022 with a diagnosis of IAI. Patients with concomitant MRSA infection from a different site or who received hemodialysis or continuous renal replacement therapy were excluded. The primary outcome was the rate of renal toxicity in patients treated empirically for IAI. Secondary outcomes included duration of treatment, duration of hospitalization, and evaluation of guideline concordant use of vancomycin in IAI.

**Results:**

Of 61 included patients, 39 (64%) were treated empirically with vancomycin for IAI. Patients on vancomycin experienced more acute kidney injury compared to those on empiric standard regimens [9/39 (23%) vs. 1/22 (5%), p=0.05]. Median duration of hospitalization for patients on vancomycin vs. standard therapy was 11 days (IQR, 14) vs. 5 days (IQR, 4), p < 0.05, respectively. Median length of inpatient therapy was 9 days (IQR, 12) vs. 4 days (IQR, 5), p < 0.05, for patients on vancomycin vs. standard therapy. Notably, non-guideline concordant use of vancomycin occurred in 14/39 (46%) patients and no patients had cultures grow MRSA.

**Table 1.**
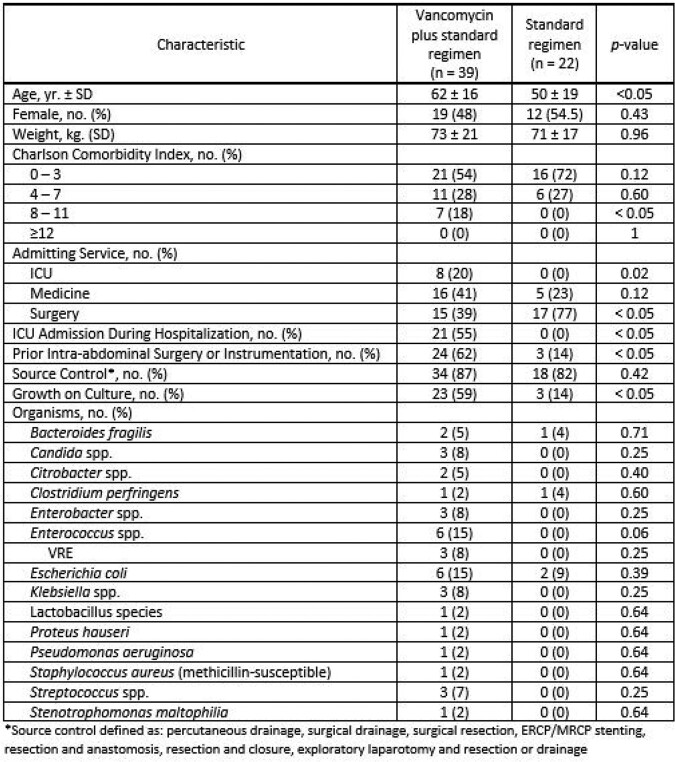
Baseline Characteristics

**Table 2.**
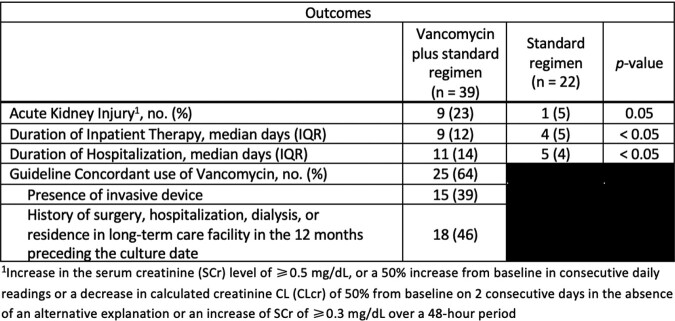
Primary and Secondary Outcomes

**Conclusion:**

More patients who were treated with vancomycin experienced acute kidney injury. Patients who were treated with standard therapy were more likely to have a reduced hospital length of stay and shorter inpatient antibiotic duration of treatment. Institutional guidelines recommend no additional coverage outside of specific risk factors but stewardship efforts do not extend to this use. Future efforts to decrease use of vancomycin for IAI may result in a decrease in observed harms. Further studies are needed to refine which patients may truly benefit from empirical vancomycin therapy.

**Disclosures:**

**All Authors**: No reported disclosures

